# The mTOR kinase inhibitor rapamycin decreases iNOS mRNA stability in astrocytes

**DOI:** 10.1186/1742-2094-8-1

**Published:** 2011-01-05

**Authors:** Lucia Lisi, Pierluigi Navarra, Douglas L Feinstein, Cinzia Dello Russo

**Affiliations:** 1 Istitute of Pharmacology, Catholic Medical School, Rome, Italy; 2 Department of Anesthesiology, University of Illinois at Chicago, Chicago, Illinois, USA

## Abstract

**Background:**

Reactive astrocytes are capable of producing a variety of pro-inflammatory mediators and potentially neurotoxic compounds, including nitric oxide (NO). High amounts of NO are synthesized following up-regulation of inducible NO synthase (iNOS). The expression of iNOS is tightly regulated by complex molecular mechanisms, involving both transcriptional and post-transcriptional processes. The mammalian target of rapamycin (mTOR) kinase modulates the activity of some proteins directly involved in post-transcriptional processes of mRNA degradation. mTOR is a serine-threonine kinase that plays an evolutionarily conserved role in the regulation of cell growth, proliferation, survival, and metabolism. It is also a key regulator of intracellular processes in glial cells. However, with respect to iNOS expression, both stimulatory and inhibitory actions involving the mTOR pathway have been described. In this study the effects of mTOR inhibition on iNOS regulation were evaluated in astrocytes.

**Methods:**

Primary cultures of rat cortical astrocytes were activated with different proinflammatory stimuli, namely a mixture of cytokines (TNFα, IFNγ, and IL-1β) or by LPS plus IFNγ. Rapamycin was used at nM concentrations to block mTOR activity and under these conditions we measured its effects on the iNOS promoter, mRNA and protein levels. Functional experiments to evaluate iNOS activity were also included.

**Results:**

In this experimental paradigm mTOR activation did not significantly affect astrocyte iNOS activity, but mTOR pathway was involved in the regulation of iNOS expression. Rapamycin did not display any significant effects under basal conditions, on either iNOS activity or its expression. However, the drug significantly increased iNOS mRNA levels after 4 h incubation in presence of pro-inflammatory stimuli. This stimulatory effect was transient, since no differences in either iNOS mRNA or protein levels were detected after 24 h. Interestingly, reduced levels of iNOS mRNA were detected after 48 hours, suggesting that rapamycin can modify iNOS mRNA stability. In this regard, we found that rapamycin significantly reduced the half-life of iNOS mRNA, from 4 h to 50 min when cells were co-incubated with cytokine mixture and 10 nM rapamycin. Similarly, rapamycin induced a significant up-regulation of tristetraprolin (TTP), a protein involved in the regulation of iNOS mRNA stability.

**Conclusion:**

The present findings show that mTOR controls the rate of iNOS mRNA degradation in astrocytes. Together with the marked anti-inflammatory effects that we previously observed in microglial cells, these data suggest possible beneficial effects of mTOR inhibitors in the treatment of inflammatory-based CNS pathologies.

## Background

Astrocyte activation has been implicated in the pathogenesis of several neurological conditions, such as neurodegenerative diseases, infections, trauma, and ischemia. Reactive astrocytes are capable of producing a variety of pro-inflammatory mediators, including interleukin-6 (IL-6), IL-1β, tumor necrosis factor-α (TNF-α), neurotrophic factors [1], as well as potentially neurotoxic compounds, like nitric oxide (NO). NO, one of the smallest known bioactive products of mammalian cells, is biosynthesized by three distinct isoforms of NO synthase (NOS): the constitutively expressed neuronal (n)NOS and endothelial (e)NOS, and the inducible (i)NOS [2]. The expression of iNOS can be induced in different cell types and tissues by exposure to immunological and inflammatory stimuli [3]. In vitro, primary astrocyte cultures express iNOS in response to cytokines such as IL-1β [4], interferon γ (IFNγ), TNFα and/or the bacterial endotoxin, lipopolysaccharide (LPS) [5,6]. Once induced, iNOS leads to continuous NO production, which is terminated by enzyme degradation, depletion of substrates, or cell death [7]. iNOS activity generates large amounts of NO (within the μM range) that can have antimicrobial, anti-atherogenic, or apoptotic actions [8]. However, aberrant iNOS induction exerts detrimental effects and seems to be involved in the pathophysiology of several human diseases [9,10].

Consistently, the expression of iNOS is tightly regulated by complex molecular mechanisms, involving both transcriptional and post-transcriptional processes [2]. At the post-transcriptional level an important mechanism of regulation is the modulation of iNOS mRNA stability that is controlled by several RNA binding proteins (RNA-BPs) [11]. These proteins bind to the iNOS mRNA and allow its interaction with the exosome, the mRNA degrading machinery [2]. Interestingly, the mammalian target of rapamycin (mTOR) kinase modulates the activity of some of the above mentioned RNA-BPs [12,13] mTOR is a serine-threonine kinase that plays an evolutionary conserved role in the regulation of cell growth, proliferation, survival, and metabolism, as well as of other physiological processes such as transcription, mRNA turnover and protein translation [14]. Within the cells, mTOR can exist in at least two distinct complexes together with different partners, mTORC1 and mTORC2. The mTORC1 consists of the regulatory-associated protein of mTOR, Raptor, and the adaptor protein mLST8/GβL (G protein β-subunit-like protein), and regulates several functions related to cell cycle and growth. The mTORC2 includes mLST8, the adaptor protein Rictor, and Sin1 [15]. mTORC2 is thought to regulate the actin cytoskeleton dynamics [16]. Indeed, rapamycin is a second generation immunosuppressant drug that blocks T-cell proliferation by inhibition of mTOR activity, and it is normally used to prevent transplant rejection in association with the older calcineurin inhibitors [17] mTORC1 activity is inhibited by rapamycin and its analogs, while mTORC2 is insensitive to the rapamycin inhibitory actions at least at immunosuppressive concentrations [18].

mTOR is also a key regulator of intracellular processes in glial cells, in fact various mTOR upstream regulators have been reported to play an important role in astrocyte physiology. For example, inactivation of a negative regulator of the mTOR pathway, the tumor suppressor PTEN, promotes astrocyte hypertrophy and proliferation [14]; inactivation of the tumor suppressor tuberin leads to glial cell hypertrophy and formation of glial hamartomas [14]; up-regulation of mTOR signaling regulates glutamate transporter 1 [19]; and down-regulation of mTOR/S6 kinase pathway contributes to astrocyte survival during ischemia [20]. We recently showed that rapamycin and its analog, RAD001, reduce iNOS expression and activity in microglial cultures activated by pro-inflammatory cytokines while displaying minor effects on astrocyte iNOS [21], thus suggesting that mTOR regulates glial inflammatory activation but with selective effects. However, with respect to iNOS expression, both stimulatory and inhibitory actions involving the mTOR pathway have been described. In particular, rapamycin has been found to down-regulate LPS-induced iNOS protein expression in the mouse macrophage RAW 264.7 cell line via mTOR dependent proteasomal activation [22]. In contrast, TNF-α increases iNOS expression and NO production in myoblasts via the activation of the ILK/Akt/mTOR and NFkB signaling pathway [23]; and ultra-sound stimulation up-regulates iNOS expression by an HIF-1α-dependent mechanism involving the activation of ILK/Akt and mTOR pathways via integrin receptor in osteoblasts [24]. Thus, mTOR differentially regulates iNOS activity and expression depending on the cell type or activating stimulus, and this may explain the different effects that we observed on glial iNOS [21].

In our previous studies, we observed that although rapamycin reduced iNOS expression mRNA and activity in microglial cells, and was without effect on astrocyte iNOS activity [21], it caused a rapid significant increase in iNOS mRNA levels in astrocytes induced by two different proinflammatory stimuli. Later time points were not examined; neither was the basis for this contrasting result examined. In the present paper we tested the hypothesis that while at early times rapamycin increases iNOS mRNA, at later times it modifies iNOS mRNA stability. Our results using primary rat astrocytes are consistent with this hypothesis, and suggest that inhibition of mTOR kinase activity in glial cells results in anti-inflammatory actions. Together with the marked anti-inflammatory effects observed in microglial cells [21], these data further provide pre-clinical evidence for a possible clinical use of mTOR inhibitors in the treatment of inflammatory-based CNS pathologies.

## Methods

### Materials

Cell culture reagents [Dulbecco's modified Eagle's medium (DMEM), DMEM-F12 and Fetal calf serum (FCS)] were from Invitrogen Corporation (Paisley, Scotland). Antibiotics were from Biochrom AG (Berlin, Germany). Bacterial endotoxin LPS (Salmonella typhimurium) was from Sigma-Aldrich (St. Louis, MO, USA). Recombinant pro-inflammatory cytokines, namely human tumor necrosis factor α (TNFα), human interleukin-1β (IL-1β) and rat interferon-γ (IFNγ) were purchased from Endogen (Pierce Biotechnology, Rockford, IL, USA). Rapamycin (RAPA) was purchased from Tocris Bioscience (Bristol, UK). β-actin (clone AC-74) mouse monoclonal antibody was from Sigma Aldrich; rabbit polyclonal anti-phospho [ser-2448] mTOR was purchased from Novus Biological (Littleton, CO, USA), mouse monoclonal iNOS antibody was from Santa Cruz (Santa Cruz, CA).

### Cell cultures

Primary cultures of rat cortical astrocytes were prepared as previously described [25]. In brief, 1- to 2-day-old Wistar rats were sacrificed. The brains were removed under aseptic conditions and placed in phosphate buffer saline with Ca^++ ^and Mg^++ ^(PBS-w), containing antibiotics (100 IU/ml of penicillin plus 100 μg/ml streptomycin). Under a stereomicroscope, the meninges were carefully removed and the cortex was dissected. The tissue was cut into small fragments, digested with trypsin in PBS without Ca^++ ^and Mg^++ ^(PBS-wo), for 25 min at 37°C and for further 5 min in the presence of DNAse I. This step was followed by mechanically dissociation in Dulbecco's MEM with Glutamax-I containing 10% fetal calf serum (FCS) and antibiotics as above, to obtain single cells. Cell viability was roughly 45%.

The cells thus obtained were seeded in 75-cm^2 ^flasks at a density of 1 × 10^7 ^cells/10 ml (1 brain/flask) and incubated at 37°C in a humidified atmosphere containing 5% CO2. The medium was changed after 24 h and then twice a week. Astrocytes obtained with this procedure were then subcultured twice, the first time in 75-cm^2 ^flasks and the second time directly in multiwell plates used for the experimental procedures. All the experiments were carried out in 1% FCS DMEM with antibiotics.

C6 glioma cells and stable transfected C6 cells (see below) were grown in DMEM containing 10% fetal calf serum and antibiotics, including G418. Cells were passed once a week and used for the experiments after 3-4 days, when they reached 90% confluence.

### Nitrite assay

NOS2 activity was assessed indirectly by measuring nitrite accumulation in the incubation media. Briefly, an aliquot of the cell culture media (80 μL) was mixed with 40 μL Griess Reagent (Sigma Aldrich) and the absorbance measured at 550 nm in a spectrophotometric microplate reader (PerkinElmer Inc., MA, USA). A standard curve was generated during each assay in the range of concentrations 0-100 μM using NaNO_2 _(Sigma Aldrich) as standard. In this range, standard detection resulted linear and the minimum detectable concentration of NaNO_2 _was ≥ 6.25 μM.

In the absence of stimuli, basal levels of nitrites were below the detection limit of the assay after 24 and 48 h incubations.

### iNOS promoter luciferase assay

C6-2.2. cells stably transfected with a 2,168-bp fragment of the rat iNOS promoter driving luciferase expression [26] were used to monitor effects of rapamycin on activation of the iNOS promoter. These cells have a low level of basal luciferase activity, which can be induced between 4- and 10-fold upon incubation with LPS plus IFNγ or with TII. C6-2.2. cells were incubated with the indicated iNOS inducers in DMEM containing 1% FCS and the indicated concentrations of rapamycin. After desired incubation times, the media were removed, and the cells were washed once with cold phosphate-buffered saline. To prepare lysates, 50 μl of CHAPS buffer (10 mM CHAPS, 10 mM Tris, pH 7.4) were used. Aliquots of cell lysates (40 μl) were placed into wells of an opaque white 96-well microplate. A volume of luciferase substrate (20 μl) (Steady Glo reagent, Promega) was added to all samples, and the luminescence was measured in a microplate luminometer (Rosys-Anthos, Austria). The data are presented as the percentage of luciferase activity measured in the presence of NOS2 inducers and rapamycin, relative to the activity of control cells (incubated in media with TII).

### iNOS mRNA analysis in real time PCR

Total cytoplasmic RNA was extracted from astrocytes using TRIZOL (Invitrogen). RNA concentration was measured using the Quant-iT™ RiboGreen^® ^RNA Assay Kit (Invitrogen). In each assay, a standard curve in the range of 0-100 ng RNA was run using 16S and 23S ribosomal RNA (rRNA) from E. coli as standard. Aliquots (1 μg) of RNA were converted to cDNA using random hexamer primers and the ImProm-II Reverse Transcriptase (Promega, Madison, WI, USA). Quantitative changes in mRNA levels were estimated by real-time PCR (Q-PCR) using the following cycling conditions: 35 cycles of denaturation at 95°C for 20 s; annealing at 59°C for 30 s; and extension at 72°C for 30 s; Brilliant SYBR Green QPCR Master Mix 2× (Stratagene, La Jolla, CA, USA) was used. PCR reactions were carried out in a 20 μL reaction volume in a MX3000P real time PCR machine (Stratagene). The primers used for iNOS detection were: 1704F (50-CTG CAT GGA ACA GTA TAA GGC AAA C-30), and 1933R (50-CAG ACA GTT TCT GGT CGA TGT CAT GA-30), which yield a 230 base pair (bp) product. The primers used for α-tubulin were: F984 (50-CCC TCG CCA TGG TAA ATA CAT-30), and 1093R (50-ACT GGA TGG TAC GCT TGG TCT-30), which yield a 110 bp product. The primers used for TTP detection were: 2F (5'- CAG CCT GAC TTC TGC GAA CCG A -3'), 102R (5'- TGG CTC ATC GAC ATA AGG CTC TCG T -3'), which yield a 101 base pair (bp) product. The primers used for KSRP were: 202F (5'- CCG GGG ATA CGC AAG GAC GC -3'), 472R (5'- CCA CCA TGC CGT CCG GAA CC -3'), which yield a 271 bp product. The primers used for HuR detection were: 512F (5'- AAC CCC CGG GTT CCT CCG AG -3'), 727R (5'- CCG AGG AAG CAT TGC CGG GG -3'), which yield a 216 base pair (bp) product. Relative mRNA concentrations were calculated from the take-off point of reactions (threshold cycle, Ct) using the comparative quantization method and the software included in the unit. For this analysis, controls were used as calibrators and the Ct values for α-tubulin expression as normalizers. Thus, using the -ΔΔCt method, we calculated the differences (fold changes) in the expression of NOS2 target gene after a specific treatment *vs*. its respective control [27]. Moreover, in each run we calculated the PCR efficiency using serial dilution of one experimental sample; efficiency values were found between 94 and 98% for each primer set [21].

To test the hypothesis that rapamycin could be influencing iNOS mRNA stability, cells were incubated with TII or TII plus rapamycin for 6 h. Then, 4 μg/ml actinomycin D was added and RNA was prepared at time points from 0 to 4 h thereafter. Relative iNOS and α-tubulin mRNA amounts were determined by Q-PCR and iNOS mRNA was normalized *versus *α-tubulin mRNA. The relative amount of iNOS mRNA at 0 h was taken as 100%, and the amount at later time point reported as percentage of the 0 h time point.

### Western immunoblot

Astrocytes were lysed in RIPA buffer (1 mM EDTA, 150 mMNaCl, 1% igepal, 0.1% SDS, 0.5% sodium deoxycholate, 50 mM Tris-HCl, pH 8.0) (Sigma-Aldrich) containing protease inhibitor cocktail diluted 1:250 (Sigma-Aldrich). The protein content in each sample was determined by Bradford's method (Biorad, Hercules, CA, USA) using bovine serum albumin as standard. A 20 μg aliquot of protein was mixed 1:2 with 2× Laemmli Buffer (Biorad), boiled for 5 min, and separated through 10% polyacrylamide SDS gels. Apparent molecular weights were estimated by comparison to colored molecular weight markers (Sigma Aldrich). After electrophoresis, proteins were transferred to polyvinylidene difluoride membranes by semi-dry electrophoretic transfer (Biorad). The membranes were blocked with 10% (w/v) low-fat milk in TBST (10 mM Tris, 150 mMNaCl, 0.1% Tween-20, pH 7.6) (Biorad) for 1 h at room temperature, and incubated in the presence of the primary antibody overnight with gentle shaking at 4°C. Primary antibodies for phosphorylated mTOR, β-actin were used at the final concentration of 1:1000; primary antibody for iNOS were used at the final concentration 1:400. Primary antibodies were removed, membranes washed 3 times in TBST, and further incubated for 1h at room temperature in the presence of specific secondary antibody, anti-rabbit IgG-HRP conjugated (Vector Laboratories, Burlingame, CA, USA) diluted 1:15,000-20,000 or anti-mouse IgG-HRP conjugated (Sigma-Aldrich) secondary antibody, diluted 1:20,000. Following three washes in TBST, bands were visualized by incubation in ECL reagents (GE Healthcare) and exposure to Hyperfilm ECL (GE Healthcare NY, USA). The same membranes were washed 3 times in TBST, blocked with 10% (w/v) low-fat milk in TBST for 1 h at room temperature and used for β-actin immunoblot. Band intensities were determined using ImageJ software (National Institutes of Health) from autoradiographs obtained from the minimum exposure time that allow band detection, and background intensities (determined from an equal-sized area of the film immediately above the band of interest) were subtracted.

### Data analysis

All experiments were done using 5-6 replicates per each experimental group, and repeated at least 3 times. For the RNA analysis, samples were assayed in triplicates, and the experiments were repeated at least twice. Data were analyzed by one- or two-way ANOVA followed by Bonferroni's post hoc tests or by unpaired t-test P values < 0.05 were considered significant.

## Results

### Cytokine dependent mTOR activation does not significantly affect astrocyte iNOS activity

**Figure 1:**
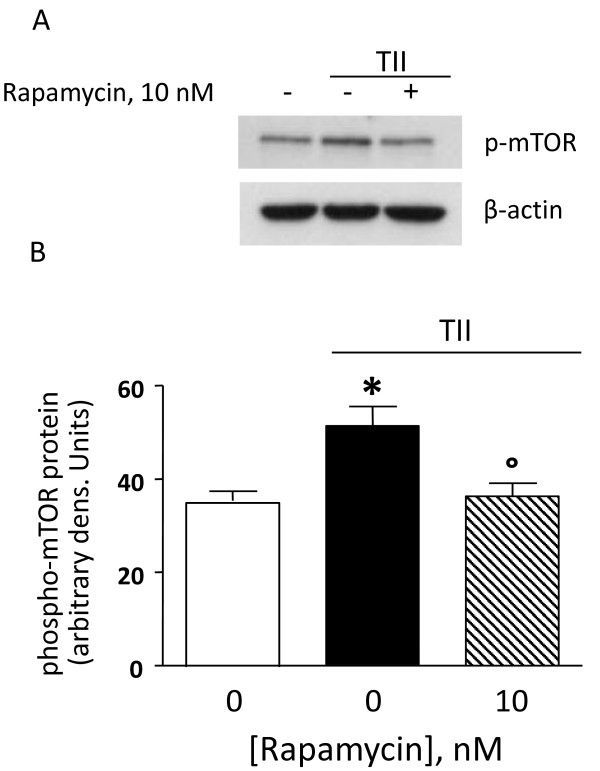
**Rapamycin reduces mTOR phosphorylation induced by TII**. (A) Whole-cell lysates were prepared from astrocytes activated with TII for 2 h. 10 nM Rapamycin was added at the beginning of the experiment and equal amounts of protein were analyzed by western blot for phosphorylated mTOR kinase (p-mTOR) (upper gel) and consequently for β-actin (lower gel). (B) Quantitation of densitometry wherein p-mTOR values are reflected relative to those for β-actin. Data are representative of two different experiments. Results were analyzed by one-way ANOVA followed by Bonferroni's post-test. *P < 0.05 vs. Controls; °P < 0.05 vs. TII.

**Figure 2:**
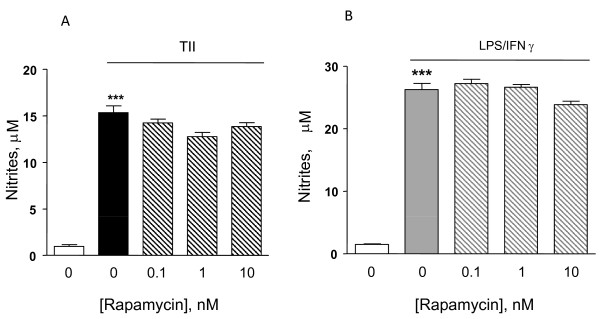
**Effects of the mTOR inhibitor on NO production in activated astrocytes**. Astrocytes were activated for 48 h with TII [Figure 2A] or 24 h with LI [Figure 2B]. The mTOR inhibitor, rapamycin was added to the cells in nM concentrations at the beginning of the experiments. NO production was assessed indirectly by measurement of nitrite accumulation in the incubation medium (Griess method). Data are expressed as means ± SEM (n = 6). Data are representative of 3 different experiments. Results were analyzed by one-way ANOVA followed by Bonferroni's post-test. ***P < 0.001 vs. Controls.

Astrocytes were stimulated using a mixture of proinflammatory cytokines (TII), i.e. 10 ng/ml IL1β, 10 ng/ml TNFα, 5 ng/ml IFNγ, and the activation of the mTOR pathway was examined by measurement of the phosphorylation level of mTOR at Ser2448 [21]. Cells were incubated for 2 h, and then subjected to protein analysis for phospho-mTOR and β-actin. TII significantly increased mTOR phosphorylation at Ser2448, which was completely blocked by10 nM rapamycin (Figure 1). Significant amounts of nitrites, a stable end product of NO, could be measured after 48 h incubation and were significantly increased by TII [21]. However, while rapamycin significantly reduced iNOS expression and activity in microglial cells, it displayed only minor effects on TII stimulated astrocytes [[21], Figure 2A]. In particular, rapamycin within the 0-10 nM range tended to reduce TII dependent NO production, but this effect did not reach statistical significance (Figure 2A). Similarly, rapamycin failed to inhibit NO production when astrocytes were exposed to another pro-inflammatory stimulus that is more widely used in vitro to activate glial cells, namely 1 μg/ml LPS and 5 ng/ml IFNγ (LI) (Figure 2B).

**Figure 3:**
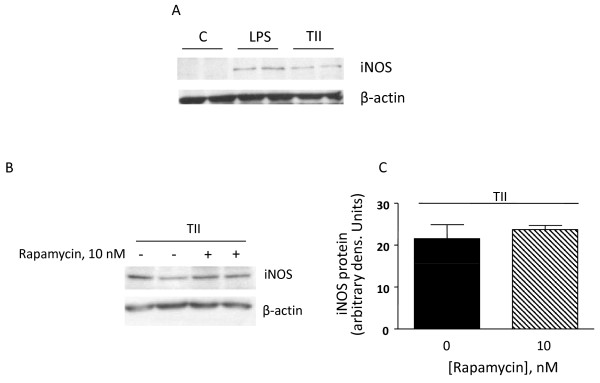
**Rapamycin does not modify the level of iNOS protein**. (A) Whole-cell lysates were prepared from Control astrocytes or cells activated with TII or LPS for 24 h. Equal amounts of proteins were analyzed by western blot for iNOS (upper gel) and consequently for β-Actin (lower gel). Two replicates per each experimental group are shown in the gel. (B) Pro-inflammatory cytokines (TII), and/or 10 nM Rapa were added at the beginning of the experiment and proteins were analyzed as described above. (C) Quantification of densitometry wherein iNOS values are reflected relative to those for β-actin. Data are representative of two different experiments.

The modest effects of rapamycin on iNOS activity were confirmed by western blot analysis. Under basal conditions, astrocytes do not express iNOS, but protein levels are significantly increased by 24 h stimulation in presence of either TII or LPS (Figure 3A). However, protein levels of iNOS measured after 24 h treatment with TII alone or in presence of 10 nM rapamycin were similar (Figure 3B, C). Together these data confirm our previous findings that in contrast to microglia cells, rapamycin has little effect on iNOS protein expression or activity.

### The mTOR pathway is involved in the regulation of iNOS expression

**Figure 4:**
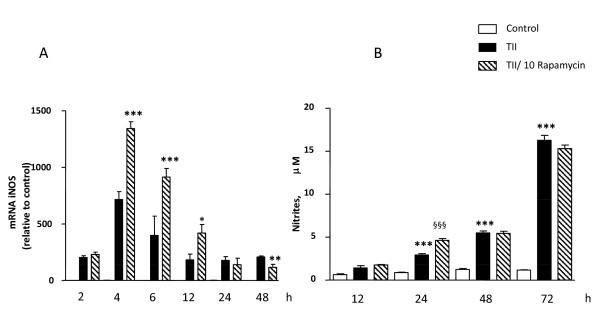
**Effects of the mTOR inhibitor on iNOS expression elicited by TII**. (A) Total cytosolic RNA was prepared from Control, or astrocytes treated with TII and rapamycin for different times, and used for Q-PCR analysis of iNOS expression. Data are expressed as fold change vs. each respective Control, taken as calibrator for comparative quantitation analysis of mRNA levels. Each sample was measured in triplicate, the experiment was repeated 2 times with similar results. Where indicated, 10 nM Rapamycin was added at the beginning of the experiment. In all time points TII significantly induced iNOS expression in comparison to Controls and rapamycin increases in 4 h the iNOS mRNA expression elicited by TII. Data are means ± SEM (n = 3). *P < 0.05, **P < 0.01, and *** P < 0.001 vs. TII; two-way ANOVA followed by Bonferroni's post-test. (B) Nitrite production elicited by TII was assessed at different time points, 10 nM rapamycin was added together with cytokines at beginning of experiment. Data are means ± SEM (n = 4). **P < 0.01, vs. Controls; and ^§§^P < 0.01, vs. TII; two-way ANOVA followed by Bonferroni's post-test.

**Figure 5:**
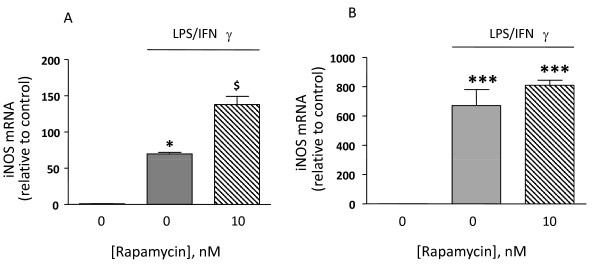
**Effects of the mTOR inhibitor RAPA on iNOS expression elicited by LI**. Total cytosolic RNA was prepared from Control, or astrocyte cells treated LI and rapamycin for different times, and used for Q-PCR analysis of iNOS expression. Data are expressed as fold change vs. each respective Control, taken as calibrator for comparative quantitation analysis of mRNA levels. Astrocytes were treated for 4 h (A) or 24 h (B). Each sample was measured in triplicate, the experiment was repeated 2 times with similar results. Where indicated, 10 nM Rapamycin was added at the beginning of the experiment. LI significantly induced iNOS expression in comparison to Controls and rapamycin increases in 4 h the iNOS mRNA expression elicited by LI. Data are means ± SEM (n = 3). ^§§^P < 0.01 vs. LI *P < 0.05 *vs*. Controls; one-way ANOVA followed by Bonferroni's post-test.

The steady state levels of iNOS mRNA were almost undetectable by Q-PCR under basal conditions (average Ct≈31), and increased in response to TII, with statistically significant increases observed after 2 h incubation, maximum levels detected after 4 h, and persistent elevation up to 48 h (the latest time point analyzed, (Figure 4A). Under these conditions, 10 nM rapamycin significantly increased iNOS mRNA levels between 4 h and 12 h incubation (Figure 4A). However, the stimulatory effect of rapamycin was transient, since no differences in iNOS mRNA were detected after 24 h between cells treated with TII and cells co-incubated with rapamycin; while after 48 h rapamycin caused significant reduction in iNOS mRNA levels (Figure 4A). The up-regulation of iNOS mRNA due to rapamycin did not directly translate into increased iNOS activity, since only a slight increase in nitrite production was measured in astrocytes co-treated with TII and 10 nM rapamycin for 24 h compared to TII alone (Figure 4B). Similarly, 10 nM rapamycin significantly increased the stimulatory effect of LI on iNOS mRNA after 4 h (Figure 5A), an effect lost after 24 h incubations (Figure 5B). Rapamycin alone did not have any significant stimulatory effect per se on either iNOS activity or on its expression (data not shown).

**Figure 6:**
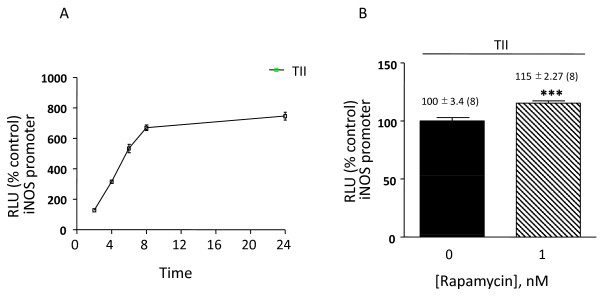
**Rapamycin increases the iNOS promoter in transfected C6 cells**. C6 cells were stable transfected with a 2,168-bp fragment of the rat iNOS promoter. (A) The cells were treated with TII or with LPS/IFNγ for indicated time point and after varying the incubation times the luciferase activity was measured. (B) Cells were incubated with TII alone or with TII/1 nM Rapamycin for 8 h. Data are presented as the percentage of luciferase activity measured in the presence of TII and rapamycin relative to the activity of TII (taken as 100%). Data are means ± SEM of n = 4 separate cell cultures; results were analyzed by unpaired t-test. ***p < 0.01 vs. TII.

**Figure 7:**
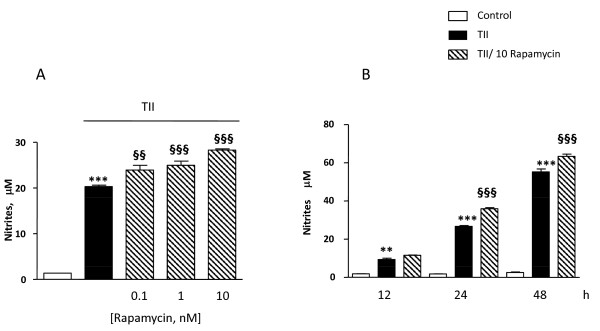
**Effects of rapamycin on NO production in activated C6 glioma cells**. (A) C6 glioma cells were activated with TII for 24 h. The mTOR inhibitor, rapamycin was added to the cells in nM concentrations at the beginning of the experiments. (B) Cells were treated with TII in presence or not of 10 nM rapamycin for different times. NO production was assessed indirectly by measurement of nitrite accumulation in the incubation medium (Griess method). Data are expressed as means ± SEM (n = 6). Data are representative of 3 different experiments. Results were analyzed by one-way (A) or two-way (B) ANOVA followed by Bonferroni's post-test. ***P<0.001 vs. Controls, and ^§§^P < 0.01, ^§§§^P < 0.001 *vs*. TII.

Extensive characterization of glial iNOS expression and regulation has been carried out with the rat C6 glioma cell line, which shares many properties with primary astrocyte cultures, including expression and regulation of the iNOS gene, mRNA and protein [6,28]. Using C6 cells stably transfected with a 2.2 kB rat iNOS promoter, we observed that TII increased the promoter activity of iNOS, with a maximum signal detected after 8 h incubation. Promoter activation lasted up to 24 h, the longest time point studied (Figure 6A). Thus, the 8 h time point was chosen for subsequent studies carried out to characterize the effects of rapamycin on iNOS promoter activity. For this, rapamycin was added at the beginning of the experiment, and after 8 h cells were washed with cold PBS and luciferase activity measured. Rapamycin, even at 10 times lower concentration, significantly increased iNOS promoter activity in comparison to cells treated with TII alone (Figure 6B). This increase of iNOS promoter activity elicited by the mTOR inhibitor may help account for the stimulatory effects of rapamycin on iNOS mRNA levels observed at earlier time points in astrocytes. However, in contrast to primary astrocytes, rapamycin dose and time-dependently increased nitrite production in C6 cells (Figure 7A, B), which suggests that additional regulatory factors are induced in astrocytes which restrict iNOS activity.

### mTOR kinase regulates the rate of iNOS mRNA degradation

**Figure 8:**
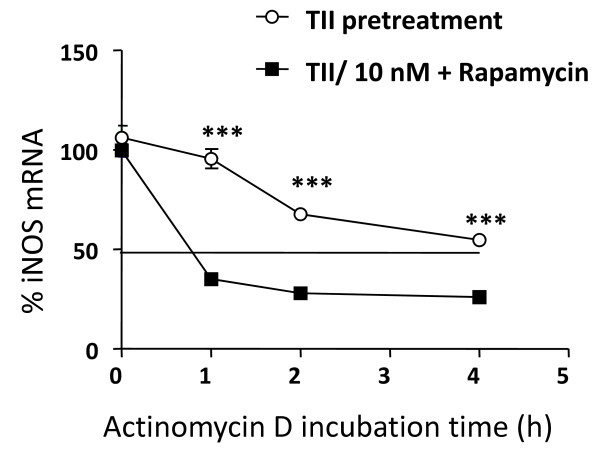
**The mTOR kinase blocking increases the rate of iNOS mRNA degradation**. Astrocytes were preincubated with TII alone or with TII plus 10 nM rapamycin for 6 h. After the pretreatment, actinomycin D (4 μg/ml) was added for different periods of time, as indicated. Cells were harvested at designated time points, total RNA was extracted and the iNOS expression was measured by real time PCR. The mRNA level at 0 time point (before the treatment with actinomycin D) was considered as 100%. Data are representative of 3 independent experiments. Data are means ± SEM (n = 3). ***P < 0.05 *vs*. TII/Rapa 10 nM; one-way ANOVA followed by Bonferroni's post-test.

**Figure 9:**
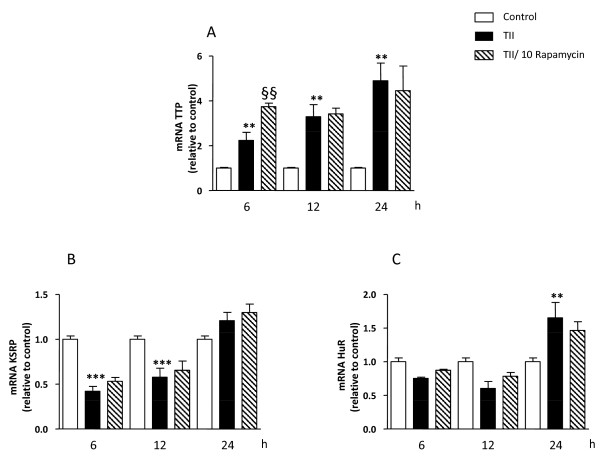
**Regulation of the expression of RNA-binding proteins by rapamycin in astrocytes**. Total cytosolic RNA was prepared from Control, or astrocyte cells treated TII and rapamycin for different times, and used for Q-PCR analysis of iNOS expression. Data are expressed as fold change vs. each respective Control, taken as calibrator for comparative quantitation analysis of mRNA levels. Astrocytes were treated for 6-12-24 h and TTP (A), KSRP (B) and HuR (C) were assessed. Each sample was measured in triplicate, the experiment was repeated 2 times with similar results. Where indicated, 10 nM Rapamycin was added at the beginning of the experiment. Data are means ± SEM (n = 3). ^§§^P < 0.01 vs. TII **P < 0.01 or ***P < 0.001 *vs*. Controls; two-way ANOVA followed by Bonferroni's post-test.

The above results suggested that rapamycin could be influencing iNOS mRNA stability. To test this possibility, the levels of iNOS mRNA were quantified in primary astrocytes after different times in the presence of TII alone or with actinomycin D added after 6 hr incubation with TII (Figure 8). The half-life of iNOS mRNA after TII treatment was determined to be 4 h, and was significantly reduced to less than 1 hour by the presence of rapamycin (Figure 8). Moreover, after 6 hr incubation in TII, rapamycin significantly increased the expression of tristetraprolin (TTP), a protein involved in iNOS mRNA stabilization (Figure 9A). In contrast, rapamycin did not modify levels of two other proteins that regulate mRNA stability, namely the KH-type splicing regulatory protein (KSRP) (Figure 9B) and HuR (Figure 9C).

## Discussion

In the present study we studied the involvement of the mTOR pathway during pro-inflammatory activation of rat cortical astrocytes in response to two different pro-inflammatory stimuli, namely a mixture of cytokines ("TII") and the bacterial endotoxin LPS augmented with IFNγ ("LI"). Moreover, the pharmacological inhibition of mTOR obtained using 10 nM rapamycin up-regulated iNOS mRNA levels without any relevant effect on iNOS activity. This opposite result is explained by the parallel increase in the rate of iNOS mRNA degradation associated with the inhibition of mTOR kinase. Rapamycin treatment did not interfere with astrocyte viability or proliferation, as we previously reported [21]. These findings clearly indicate that the blocking of mTOR pathway is crucial for the production of NO, and that the overall effect observed can be regarded as anti-inflammatory since the up-regulation of iNOS was compensated with increased iNOS degradation machinery activity.

The possible involvement of mTOR during pro-inflammatory astrocyte activation was studied by direct evaluation of the levels of mTOR phosphorylation at Ser2448 in presence of proinflammatory cytokines [21]. Phosphorylation at Ser2448 is a marker of mTOR pathway activation, as suggested by the finding of Rosner and collaborators that down-regulation of mTOR kinase ser2448 phosphorylation correlates with decreased mTORC1 activity [29]. In our experimental conditions, phospho-mTOR at Ser2448 was increased by TII and rapamycin completely abolished the phosphorylation, suggesting that proinflammatory activation of astrocytes involves the recruitment of the mTOR pathway in a rapamycin sensitive manner (Figure 1), suggesting that the drug can reduce astrocyte proinflammatory activation.

Usually under inflammatory conditions, high levels of NO are generated by the up-regulation of iNOS. The protein can be induced by different proinflammatory stimuli to different extents. In general, LPS is a more robust activator, thus nitrite levels (an indirect measure of iNOS activity) are significantly elevated after 24 h incubation, whereas in the presence of pro-inflammatory cytokines (TII) a significant increase in nitrite levels is generally detectable after 48 h incubation. These data may be explained by the recruitment of different signaling pathways by the two stimuli, and or by a different timing in recruitment, as happens in microglial cells [21]. Regardless of the stimulus used, rapamycin did not modify nitrite levels (Figure 2), while it was able to increase the amount of iNOS mRNA at the earlier time points. This up-regulation was transient, because such differences were absent at 24 h (Figure 4 and 5) and a significant reduction was observed at 48 h (Figure 4A). Rapamycin alone did not display pro-inflammatory effects per se, being unable to increase either iNOS mRNA levels or nitrite production after 4 and 24 h incubations (data not shown).

The expression of iNOS is tightly regulated by both transcriptional and post-transcriptional mechanisms. This includes regulation of iNOS promoter activity by binding of transcription factors (NFkB, STAT-1α) and the modulation of iNOS mRNA expression. Post-transcriptional regulation of gene expression is often dependent on sequences located in the 3'-untraslated region (3'-UTR) of mRNAs [30]. AU-rich (ARE) elements have been shown to confer destabilization of mRNAs coding for pro-inflammatory proteins or oncogenes [31]. In mammalian cells, ARE mediates mRNA decay mainly by recruitment of the exosome to the mRNAs, thereby promoting their rapid degradation. However, the mammalian exosome does not seem to recognize the ARE-containing RNAs on its own but requires certain ARE binding protein (ARE-BPs) for this interaction. In particular in untreated cells the RNA-Binding Protein (RNA-BP) KH-type splicing regulatory protein (KSRP) or the zinc-finger protein tristetraprolin (TTP) binds the iNOS mRNA 3'-UTR and recruits the exosome to the mRNA [32]. This results in rapid iNOS mRNA degradation. KSRP binds to the same ARE as HuR, another RNA-BP that oppositely stabilizes the mRNA, and both RNA-BPs compete for this binding sites [33]. After cytokine-treatment, p38 MAP kinase is activated and TTP expression is enhanced. The protein-protein interactions between TTP and KSRP compete for the same binding site in the iNOS 3'-UTR sequence, causing dislodgment of KSRP and enhanced HuR binding to the iNOS mRNA. The reduced KSRP and the enhanced HuR binding to the iNOS mRNA 3'UTR result in marked stabilization of the iNOS mRNA and thus in enhanced iNOS expression. Similarly, in our experimental model TII was found to significantly increase the levels of these RNA-BPs (Figure 9), while rapamycin transiently upregulated the expression of TTP without affecting the other proteins (Figure 9A). It has been shown that TTP may have opposite effects on mRNA stability in function of the AKT activation state [12]. Therefore, up-regulation of TTP in our experimental model may contribute either to the initial iNOS mRNA stabilization or be responsible for the subsequent increased rate of mRNA degradation. Additional experiments are required to completely address the role of TTP in iNOS mRNA regulation, but the present data strongly suggest a role for this protein. Consistent with our data, rapamycin was found to interfere with mRNA degradation in different cell types. For example in renal epithelial cells, Pallet and collaborators showed that rapamycin inhibits cell proliferation by regulating the expression and stability of cyclin D3 mRNA [34]. In fibroblasts rapamycin inhibition of G1 to S transition is mediated by an effect on cyclin D1 mRNA and protein stability [35]. Maderosian and colleagues showed that rapamycin regulates cyclin D1 and c-Myc mRNA in different types of tumor cells involving an enhancement of TTP binding activity [12]. Recently, Basu and collaborators demonstrated that the immunosuppressant drugs, cyclosporine and rapamycin, regulate VEGF mRNA stability in opposite manners. While cyclosporine enhances mRNA stability by induction of the RNA-BP, HuR, rapamycin increases the rate of degradation in renal cancer cells [13]. In summary, there is increasing evidence that rapamycin can regulate the activity of RNA-BPs, thus interfering with the stability of different mRNAs.

In our experimental paradigm, rapamycin increased the rate of iNOS mRNA degradation. In particular, using actinomycin D to block mRNA transcription, we found that after induction of iNOS mRNA by TII the degradation of iNOS mRNA occurs with a half life of about 4 h. Activation of astrocytes in the presence of rapamycin was characterized by an increased rate of degradation (t_1/2_. ≈ 50 min), which may be mediated by up-regulation of mRNA destabilizing proteins as occurs in other cell types [12,13]. The net effect of these complex actions on the regulation of iNOS mRNA result in similar levels of protein expression and in-significant differences in the amount of NO generated in TII-activated astrocytes in the presence or absence of rapamycin.

## Conclusions

Our findings suggest that rapamycin does not directly exert pro-inflammatory actions in rat primary cultures astrocytes, nor does it increase the proinflammatory effects of cytokines or LPS. In fact, the iNOS mRNA up-regulation induced by rapamycin administered together with a proinflammatory stimuli appears to be transient and accompanied by an increased rate of iNOS mRNA degradation. Such effects do not augment iNOS protein levels (Figure 3) nor the amount of nitrite production to any significant extent. Together with the marked anti-inflammatory effects observed in microglial cells [21], these data suggest possible beneficial effects of mTOR inhibitors in the chronic treatment of inflammatory-based CNS pathologies.

## Competing interests

The authors declare that they have no competing interests.

## Authors' contributions

LL carried out the experiments and has made substantial contributions to conception and acquisition of data, and contributed to draft the manuscript; PN has been involved in the revision of the manuscript and given final approval of the version to be published; DLF has been involved in revising the manuscript critically for important intellectual content; CDR has made contributions to design, analysis and interpretation of data, and drafted the manuscript. All authors have read and approved the final version of the manuscript.
